# An Ecological Assessment of *Isaria fumosorosea* Applications Compared to a Neonicotinoid Treatment for Regulating Invasive Ficus Whitefly

**DOI:** 10.3390/jof5020036

**Published:** 2019-05-04

**Authors:** Pasco B. Avery, Vivek Kumar, Edward A. Skvarch, Catharine M. Mannion, Charles A. Powell, Cindy L. McKenzie, Lance S. Osborne

**Affiliations:** 1 Institute of Food and Agricultural Sciences (IFAS), Indian River Research and Education Center, University of Florida, 2199 South Rock Road, Fort Pierce, FL 34945, USA; capowell@ufl.edu; 2 IFAS, Mid-Florida Research and Education Center, University of Florida, 2725 S. Binion Road, Apopka, FL 32703, USA; vivekuf@gmail.com (V.K.); lsosborn@ufl.edu (L.S.O.); 3 IFAS, Saint Lucie County Extension, University of Florida, 8400 Picos Road, Fort Pierce, FL 34945, USA; eask@ufl.edu; 4 IFAS, Tropical Research and Education Center, University of Florida, 18905 SW 280 Street, Homestead, FL 33031, USA; cmannion@ufl.edu; 5 ARS, U.S. Horticultural Research Laboratory, Subtropical Insect Research Unit, USDA, 2001 South Rock Road, Fort Pierce, FL 34945, USA; cindy.mckenzie@ars.usda.gov

**Keywords:** residential, enzootic, entomopathogenic fungi, biocontrol, weeping fig, *Encarsia protransvena*, *Amitus bennetti*, *Purpureocillium lilacinum*

## Abstract

A pilot study was conducted on a weeping fig, *Ficus benjamina* shrub hedge in a Florida urban landscape to determine the efficacy of a fungal biopesticide, PFR-97™ which contains blastospores of *Isaria fumosorosea*, and a neonicotinoid treatment (Admire Pro™) applied against the invasive ficus whitefly pest, *Singhiella simplex* (Singh). Post treatment, an ecological assessment of the study was conducted by observing the impact of the fungal biopesticide and neonicotinoid treatment on natural enemies, e.g., predators, parasitoids and enzootic fungal pathogens occurring in the whitefly-infested hedge. Both treatments provided a significant reduction in the whitefly population compared to control and were compatible with the natural enemies present. Various natural enemies including fungal entomopathogens were identified associated with the whitefly population infesting the weeping fig hedge. The parasitoids, *Encarsia protransvena* Viggiani and *Amitus bennetti* Viggiani & Evans combined parasitized a similar mean number of whitefly nymphs in both treatments and control; however, the number parasitized decreased over time. Natural enzootic fungi isolated from the ficus whitefly nymphs were *I. fumosorosea*, *Purpureocillium lilacinum* and *Lecanicillium*, *Aspergillus and Fusarium* species. Results from this pilot study suggest there is much potential for using repeated applications of the fungal biopesticide, PFR-97™ as a foliar spray compared to a neonicitionid as a soil drench for managing *S. simplex* on *Ficus* species for ≥28 days.

## 1. Introduction

The ficus whitefly, *Singhiella simplex* (Singh), is an invasive species that has become a major pest in Florida feeding on *Ficus* shrubs and trees [1,2]. This exotic species of whitefly endemic to the South Asian region, i.e., Myanmar, China and India [3,4,5], was first discovered in the United States in 2007 from Miami-Dade County in Florida [1], and since then it has become a problem for homeowners, residential community managers, landscapers, growers, businesses and government officials throughout the State [6]. Within a few years of its introduction, it has been reported damaging *Ficus* sp. in 16 counties of Florida [7]. Feeding by this pest not only turns host leaves yellow, but heavy infestations often lead to leaf drop, branch dieback and complete defoliation. 

In Florida, the ficus whitefly has been most commonly found infesting weeping fig (*Ficus benjamina*), but has also been seen on *F. altissima*, *F. aurea*, *F. bengalensis*, *F. lyrata*, *F. macllandii* and *F. microcarpa* [6,7]. Weeping figs are commonly used as hedges but are also grown as ornamental trees; particularly in the southern part of Florida. Due to the severity of damage and aesthetics or economic loss associated with this pest, current control strategies rely heavily on the use of chemical insecticides. Pest management professionals recommend soil or trunk applications of a neonicotinoid compound. Foliar sprays are also suggested to treat “hot spots” or to obtain quick knockdown in addition to the soil applications [6,7]. Although when applied appropriately, systemic insecticides in the neonicotinoid class can provide sufficient control of whitefly for 9–12 months; however, use of chemical insecticides cannot be ultimately considered a sustainable management approach for any pest. Risks of chemical insecticide use in urban landscape areas may include: (1) insecticide drift from foliar sprays [8], (2) leaching and runoff of insecticides into the water sources or drainage systems [9], (3) possibility of insecticide resistance development in the whitefly population due to prolonged use of the same chemical group [10,11], and (4) the negative impact on the non-target organisms, e.g. humans, domestic animals, natural enemies and pollinators [8,12,13].

In the Florida landscape, several natural enemies including enzootic entomopathogenic fungi have been observed attacking ficus whitefly and can play an important role in long-term control of this pest [2]. Awareness of these natural enemies is very important when making appropriate management decisions so as not to adversely affect them [14]. For instance, Torres-Barragán et al. [15] reported that a variety of fungal entomopathogens were responsible for managing the greenhouse whitefly, *Trialeurodes vaporariorum* (Westwood) population in the agricultural area at “El Eden” Ecological Reserve, Quintana Roo, Mexico. In another related study, Nielsen and Hajek [16] found that enzootic fungal entomopathogens were important in controlling invasive soybean aphid populations (*Aphis glycine* L.) and an epizootic of these insect pathogens was associated with the decline in the pest population. In addition, these authors reported that several species of parasitoids and predators (compatible with fungal entomopathogens) played a significant role and showed an additive effect in suppressing the aphid populations over time. Thus, the pest management strategy recommendations for ficus whitefly must be made considering its potential long-term detrimental effect on the naturally occurring biocontrol agents in the region, including fungal entomopathogens. 

Previous studies have indicated that fungal entomopathogens are important ecological regulatory factors in managing insect populations [15,16,17,18,19,20]. Some of the commercially available entomopathogenic fungi in formulated products which are frequently used for whitefly control are *Isaria fumosorosea* Wize [21,22], *Beauveria bassiana* (Balsamo) Vuillemin [23], *Lecanicillium muscarium* (Petch) Zare and Gams [24], and *Ashersonia aleyrodis* (Webber) [25,26]. In 1986, a strain of *I. fumosorosea* named Apopka 97, was discovered and isolated from *Phenacoccus* sp. (Hemiptera: Pseudococcidae) in Apopka, Florida [27], and is now registered in the USA with a tolerance-exempt residue status under the registered commercial name PFR-97^TM^ 20% WDG [= *Paecilomyces fumosoroseus* Apopka strain 97 (ATCC 20874)] by the manufacturer Certis USA, Columbia, MD. This fungus has a worldwide distribution and is efficacious against many pestiferous arthropods, especially whiteflies which is well documented [17,21,22,23,27,28,29,30] and it also has been demonstrated to be compatible with many beneficial arthropods, which include parasitoids and predators [22,29,31,32,33,34,35]. In addition to efficacy, the advantages of using fungal entomopathogens are numerous and include safety for humans and other non-target organisms, reduction of pesticide residues, preservation of other natural enemies, and an increased biodiversity in managed ecosystems [17]. The fungus grows optimally between 25–28 °C in the southern USA and it tolerates temperatures between 32–35 °C [27]. Thus, in Florida, the use of this formulated commercial product containing the fungus, *I. fumosorosea* may be an alternative for the management of the ficus whitefly in the urban landscape.

**Figure 1:**
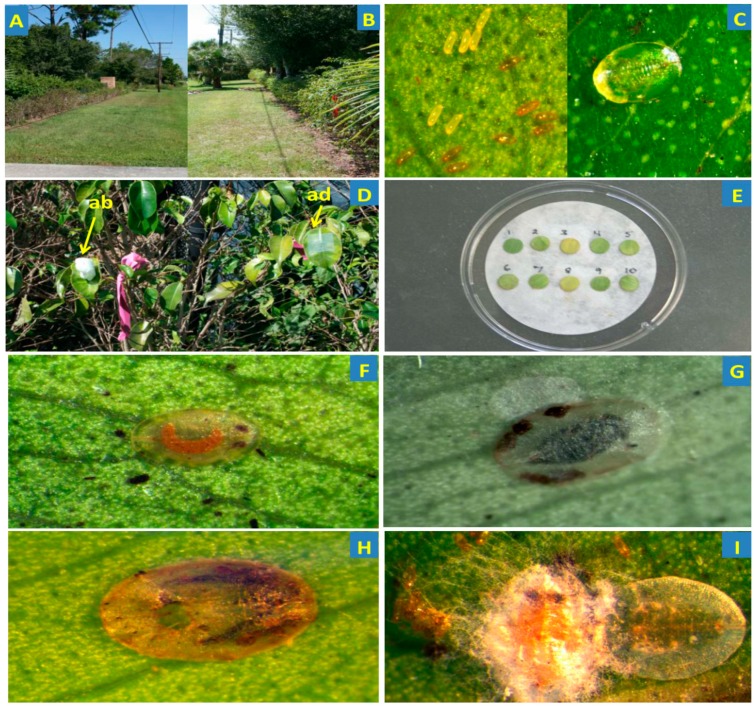
Study area and biological parameters of the study: (**A**) *Ficus benjamina* hedge view north; (**B**) view south; (**C**) eggs (left side: magnified 20×) and nymphs (right side: 16×) of the ficus whitefly found on the leaves; (**D**) Plastic coverslips pinned to either the abaxial (ab) or adaxial (ad) side of randomly chosen leaves used for spore deposition studies; (**E**) Leaf disks placed on moist filter paper in Petri dish for counting; (**F**,**G**) Recognition of parasitism of ficus whitefly nymphs by *Encarsia protransvena* (23×) and (**G**) *Amitus bennetti* with parasitoid developing inside the translucent nymphal whitefly case (31×); (**H**) ficus whitefly pupa exuviae after emergence of the parasitoid, *A. bennetti* with an exit hole (38×); (**I**) sample ficus whitefly nymph flattened and infected with a naturally occurring enzootic fungal entomopathogen, *Lecanicillium* species (31×).

A severe ficus whitefly infestation occurred in a residential community on a *F. benjamina* hedge in Fort Pierce, Florida and needed immediate attention because of the constant leaf drop (Figure 1A,B). In our efforts to support the residents, we planned a pilot study to evaluate the efficacy of the entomopathogenic fungus, *I. fumosorosea* (strain Apopka-97) contained in the formulated bioinsecticide product, PFR-97™ 20% WDG and the neonicotinoid systemic insecticide (imidacloprid) against ficus whitefly. We also, evaluated the effect of these two insecticides on the natural enemies and the naturally occurring enzootic fungal entomopathogens present in the affected area. Thus, the objective of this pilot study was to determine the potential of the fungal biopesticide, PFR-97^TM^ compared to a neonicotinoid treatment used for management of the ficus whitefly, and to assess its ecological impact on the natural enemies in an urban landscape residential field setting.

## 2. Materials and Methods

### 2.1. Study Area

The layout for the study area (northern 27′22″50.94 N × 80′22″00.22 W and southern 27′22″48.96 N × 80′22″00.25) was a randomized complete block design with four replications. Each plot measured ~5 m of a *F. benjamina* hedge (~10–15 plants) which ran along a concrete block wall (~1.2 m tall) located at a residence in Fort Pierce, Florida was ~1 m tall. Each hedge segment divided by a cement driveway was naturally infested with ficus whitefly (Figure 1C) with the northern side more severely infested than the southern. Prior to application of treatments, all plots were raked free of leaf litter and 0.2 kg of granular fertilizer (Rite Green 6–6–6; Sunniland Corporation, Sanford, FL, USA) was spread (183–243 cm in diameter) around the base of each shrub. The shrubs were watered thoroughly by a drip irrigation system as needed. Field environmental conditions throughout the study were monitored using the weather data website (www.wunderground.com).

### 2.2. Treatment Application

Treatments were as follows: *I. fumosorosea* (PFR-97^®^ 20% WDG; Certis USA, Columbia, MD) at 10^9^ colony forming units (CFUs)/g, neonicotinoid (Admire Pro™) and an untreated check (where the same volume of distilled water was sprayed. For the neonicotinoid treatment plots, each shrub was drenched at label rate with 120 mL of Admire Pro at the base by pouring the solution around each shrub. The fungal suspension was prepared by mixing the dry powder formulation of PFR-97 20% WDG approximately 2 hours prior to application in a clean bucket (18.9 L) in order to initiate the germination process and then transported to the spray site. When on site, the bucket cover was removed, and the fungal suspension was stirred again for two minutes. The suspension was then poured into a stainless-steel hand pump sprayer and the pressure was established at 30 pumps per plot. The foliage was sprayed to runoff at a rate of 2.4 g L^−1^ (2.2 × 10^7^ blastospores mL^−1^) for the 1st and 2nd application at 0 DAT and 15 DAT, respectively. The fungal suspension was applied in the evening at dusk from ~6:15–6:30 pm.

To determine the deposition of fungal blastospores mm^−2^ in the fungal treatment plots (PFR-97), five plants were chosen at random and 10 plastic microscope cover slips (Fisherbrand^®^ 22 × 22 mm, Fisher Scientific, Pittsburgh, PA, USA) were pinned to either the adaxial (5 coverslips) or abaxial (5 coverslips) side of a randomly chosen leaf per plant for the initial spray treatment (Figure 1D). The pin was made secure by sticking it into a styrofoam packing peanut on the opposite side of the leaf. On 14 DAT, prior to the second application, eight plants were chosen at random to determine the blastospore density mm^−2^ sprayed on the leaves as described above. Sprayed cover slips were allowed to dry for ~12 h overnight and then brought back to the lab for assessment of spore deposition. The pin was removed and each cover slip was placed upside down on a glass microscope slide in a 50 µL drop of acid fuschin 1% stain. Blastospore density was assessed with a compound light microscope (400×) using a 10 mm reticule grid (Hunt Optic and Imaging, Pittsburg, PA, USA). 

The viability of blastospores was assessed using two Fisherbrand^®^ Petri dishes (Thermo Fisher Scientific, Waltham, MA, USA) containing potato dextrose agar (PDA) each sprayed at a rate of 2.2 × 10^7^ spores mL^−1^. Plates were then sealed with Parafilm^®^ (Bemis, Neenah, WI, USA) and incubated for 12 h at 25 ± 1.0 °C, 100% relative humidity (RH) under a 16 h photophase. After this duration, each plate was viewed under a compound microscope and the percent viability was determined by observing a total of 200 spores (50 spores in each quadrat of the plate). Spores were considered to have germinated if a germ tube had formed. This procedure was repeated for each fungal spray application, and the percent viability ranged between 87%–89%.

### 2.3. Fungal Treatment Efficacy on Ficus Whitefly Population

Randomly chosen leaf samples (10 per plot―5 mottled and 5 new, i.e., young and fully developed) were detached 0 (pre-treatment), 7, 14, 15, 21, 28 and 35 days after initial application of treatments. A total of 10 leaf samples/plot/treatment were placed into pre-labeled individual re-sealable plastic bags and brought back to the lab for examination under a binocular microscope (40×) to record the number of live and dead whitefly nymphs. Leaf disk samples were punched out with # 5 cork borer (50.3 mm^2^) in the center of each leaf on either side of the midrib (Figure 1E). One disk was used for assessment of dead and/or parasitized ficus whitefly nymphs; the other from the same leaf was used for leaf washes (described below). Recognition of parasitism was assessed by observing the development of the parasitoid inside the translucent nymphal case, a blackened nymphal case due to melanization or an exit hole (Figure 1F–H). Once disks used for counting whitefly nymphs were observed and recorded, they were placed on moistened filter paper in a Fisherbrand^®^ Petri dish (100 × 15 mm), covered, and sealed with Parafilm^®^. Sealed dishes were then placed in a growth chamber under the same conditions as described above for 14 days to allow for mycosis and determine percent mortality due to *I. fumosorosea* and other fungal species present (if any). 

### 2.4. Fungal Identification on Leaf Phylloplane

Ten single new leaf disk/plot/treatment punched from the opposite side of the midrib (50.3 mm^2^) as described above were placed together into a 15 mL plastic centrifuge tube containing 10 mL of Triton X-100 solution (0.01%) and then vortexed for 1 minute. Aliquots (100 µL) of each suspension were removed and spread on five plastic Fisherbrand^®^ Petri dish plates (100 × 15 mm) containing PDA–dodine (modified as a selective media for entomopathogenic fungi), streptomycin sulfate and chloramphenicol [36,37]. Plates were sealed with Parafilm^®^, placed in the same growth chamber under the same conditions described above and incubated for 14 days to allow the growth of CFUs on the plates. This procedure was repeated four times for a total of 20 plates per treatment. The CFUs were used for identification of enzootic fungal entomopathogens present on the leaf phylloplane and to assess the viability of *I. fumosorosea*. Voucher fungal entomopathogen in vitro culture isolates were identified by Svetlana Gouli and deposited at the University of Vermont, Invertebrate Pathology and Microbial Pest Control Laboratory in Burlington, VT. In vitro voucher fungal entomopathogen isolates of *Isaria* species were sent to both Dr. Richard Humber at the USDA-ARS Collection of Entomopathogenic Fungal Cultures, in Ithaca, NY and Dr. Rob Samson at the CBS-KNAW Fungal Diversity Centre, Utrecht, in The Netherlands for identification and were deposited with each institution.

### 2.5. Identification of Enzootics Isolated from Ficus Whitefly

Five dead and flattened nymphs (Figure 1I) per plot/treatment were randomly chosen and removed from the semi-desiccated leaf disks using a sterile insect pin. A total of 20 cadavers per treatment from leaves collected for both treatments 0, 14 and 35 days post-treatment were placed on 1% water agar as described by Hall and Nguyen [38] in Fisherbrand^®^ Petri dish plates (100 × 15 mm). All agar plates were sealed with Parafilm, and then placed in the same growth chamber under the same conditions described above and incubated for 7 days. After mycosis of the insect was evident, the fungal spores and/or hyphae were isolated and grown on fresh PDA plates (100 × 15 mm) for identification. These inoculated plates were sealed, transferred to the growth chamber and incubated as described above. Voucher fungal entomopathogen in vitro culture isolates were identified as described above.

### 2.6. Data Analysis

The treatment effect on the total number of whitefly nymphs and percent mortality per treatment on the leaf disk per sampling day were assessed using a one-way ANOVA (α = 0.05) with mean separation by an LSD test. The percentage of nymphs on the leaf disks infected with the fungus, *I. fumosorosea* and other fungal species was determined and the number of CFUs isolated from leaf washes for *I. fumosorosea* on PDA-dodine agar plates for each treatment over time was compared. The effect of the treatments on the corrected mean percent mortality of the whitefly nymphs was determined using the Sun-Shepard’s formula [39]:

$$\begin{array}{c} \mathrm{Corrected}\;\%=\left(\frac{\mathrm{Mortality}\;\%\;\mathrm{in}\;\mathrm{treated}\;\mathrm{plot}\;\pm \;\mathrm{Change}\;\%\;\mathrm{in}\;\mathrm{control}\;\mathrm{plot}\;\mathrm{population}}{100\;\pm \;\mathrm{Change}\;\%\;\mathrm{in}\;\mathrm{control}\;\mathrm{plot}\;\mathrm{population}}\right)*100\\ \begin{array}{c} \mathrm{Change}\;\%\;\mathrm{in}\;\mathrm{control}\;\mathrm{plot}\;\mathrm{population}\\ \;=\left(\frac{\mathrm{Population}\;\mathrm{in}\;\mathrm{control}\;\mathrm{plot}\;\mathrm{after}\;\mathrm{treatment}\;-\;\mathrm{Population}\;\mathrm{in}\;\mathrm{control}\;\mathrm{plot}\;\mathrm{before}\;\mathrm{treatment}}{\mathrm{Population}\;\mathrm{in}\;\mathrm{control}\;\mathrm{plot}\;\mathrm{before}\;\mathrm{treatment}}\right)*100 \end{array} \end{array}$$

The effect of treatments on the percent parasitism was assessed and compared using an ANOVA (α = 0.05) with mean separation by a Tukey’s HSD test. Data was square root (*n* + 0.01) arcsine transformed to remove zeros prior to analysis and untransformed numbers are presented in the table. The total percent mortality of the whitefly nymphs due to fungal entomopathogens plus other biotic and/or abiotic factors and parasitization in the different treatment plots was determined and compared over time. All statistical analyses were conducted using SAS 9.4 for WINDOWS 2012 (Cary, NC).

## 3. Results

### 3.1. Field Environmental Conditions

#### 3.1.1. Pre-Treatment

During the first application of PFR-97, ambient temperature was 31 °C with a dew point of 20 °C and wind was calm with an occasional slight SW breeze; RH was 52%, and partly cloudy. The humidity increased steadily overnight reaching 78% RH by midnight with a temperature of 31 °C; RH increased to 93% by morning and temperature decreased to a low of 21 °C. During the second application the temperature was 28 °C with a dew point of 23 °C. Again, the wind was calm, with an occasional ENE breeze; RH was 76%, and partly cloudy. The humidity increased steadily overnight reaching 84% RH by midnight with a temperature of 26 °C; RH increased to 100% by morning (5:43am) and temperature decreased to a low of 22 °C.

#### 3.1.2. Post-Treatment

The humidity reached a high of 100% on days 9–11, 14–15, 18, 27, 30–31, and 34 and a low of 39% on day 0 post-treatment. The overall mean ± SEM humidity was 78 ± 2% for the duration of the study. The highest air temperature (34 °C) occurred on days 0, 6, 8, 15–16 and the lowest air temperature (8 °C) occurred on day 19 post-treatment; the overall mean ± SEM air temperature was 25 ± 0.5 °C for the duration of the study. A total of 30 mm of rainfall occurred during the duration of the study and the highest (11 mm) and second highest (10 mm) level occurred 7 and 34 days post-treatment, respectively. Rainfall of ≤ 2 mm was intermittent and occurred on days 10, 13, 17, 21–23, 26 and 35 post-treatment. The overall mean ± SEM total amount of rainfall for the duration of the study was 0.8 ± 0.4 mm.

### 3.2. Insect Pests and Natural Enemies

The insect pests observed infesting the *F. benjamina* hedge included the ficus whitefly, *S. simplex*, another whitefly infesting ficus in Florida *Tetraleurodes fici* Quaintance & Baker and the weeping ficus thrips, *Gynaikothrips uzeli* Zimmerman (Table 1).

Various natural enemies were identified managing the ficus whitefly populations that infested the weeping fig. The parasitoids, *Encarsia protransvena* Viggiani and *Amitus bennetti* Viggiani & Evans were observed after parasitization or emergence from the whitefly pupal case (Figure 1F–H). The lady beetles, *Curinus coeruleus* Mulsant, and *Harmonia axyridis* (Pallas) were observed roaming on the leaves of the *F. benjamina* hedge, and eggs and larvae of the green lacewing, *Chrysopid* sp. were observed on the leaves from all plots. The natural enzootic populations of entomopathogenic fungi inhabiting the leaf phylloplane and/or infecting the ficus whitefly were identified as follows: *I. fumosorosea*, *Purpureocillium lilacinum* (Thom) Luangsa-ard, Hou-braken, Hywel-Jones & Samson; *Lecanicillium* (Figure 1I), *Fusarium*, and *Aspergillus* species. 

### 3.3. Blastospore Deposition

The mean ± SEM spore deposition of *I. fumosorosea* blastospores mm^−2^ for the initial spray application was higher for the adaxial (391 ± 63) compared to the abaxial (168 ± 45) side of the leaves; the mean deposition per leaf was 280 ± 43. For the second spray application, the mean ± SEM spore deposition of *I. fumosorosea* blastospores mm^−2^ was again higher on the adaxial side (376 ± 135) compared to the abaxial side (215 ± 44); mean deposition on each leaf was 295 ± 72 blastospores mm^−2^.

### 3.4. Population Density of and Treatment Effects on Whitefly Nymphs

**Figure 2:**
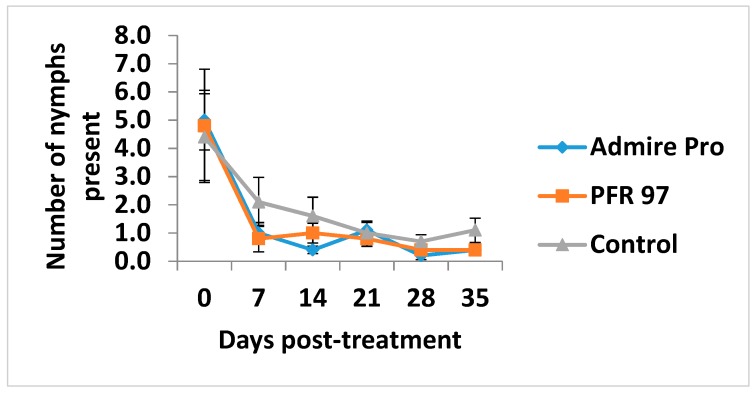
Mean number ± SEM of live ficus whitefly nymphs observed on the leaf disks pre-treatment and over time for the duration of the study. Error bars at each day pre- and post-treatment per treatment represent the ± SEM of the number of nymphs present. The number of nymphs observed at each day post-treatment were not significantly different amongst the treatment plots (Tukey’s HSD test, *p* > 0.05).

The mean number ± SEM of live nymphs observed on the leaf disks pre-treatment (day 0) was not significantly different (*p* > 0.05) for PFR-97 (4.8 ± 2.0), Admire Pro (5.0 ± 1.0) and control (4.4 ± 1.5) (Figure 2).

The mean number of nymphs observed on the leaf disks pre-treatment and for the duration of the study were not significantly different amongst the treatments. The number of nymphs decreased in number and showed a downward trend over time. 

**Figure 3:**
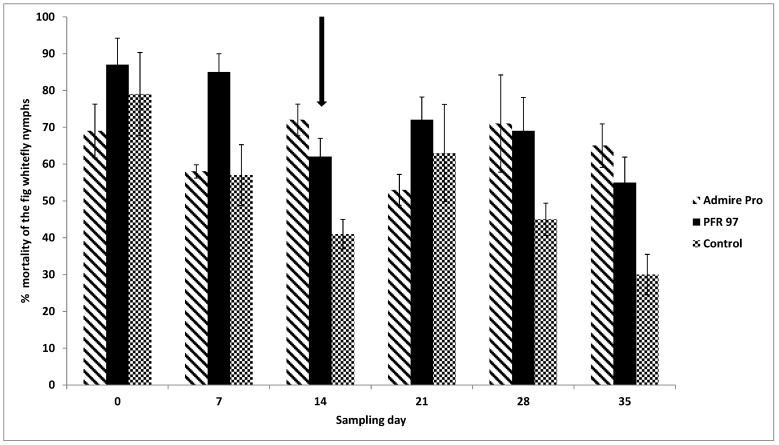
Effect of the fungal biopesticide PFR-97, neonicotinoid Admire Pro on the percent mortality of the ficus whitefly nymphs observed on *Ficus benjamina*. Data were arc sine transformed prior to analysis; untransformed data are presented. Bars represent percent mortality ± SEM. Mean numbers are based on observing 40 leaf disks per treatment. Arrow indicates when PFR-97 was applied a second time on day 14. Letters above the bars per sampling day that are different indicates significance amongst the treatments (LSD test, *p* < 0.05).

The mean percent mortality of the ficus whitefly nymphs 7 days post-treatment after the 1st PFR-97 application was higher compared to the control treatment (*F* = 5.16; df = 2, 6; *p* < 0.05) (Figure 3). 

On day 14, nymphal mortality for both treatments were significantly higher (*F* = 14.4; df = 2, 9; *p* = 0.005) compared to the control. There were no significant differences in percent mortality for the next 2 weeks (*F* = 0.80; df = 2, 6; *p* = 0.493; *F* = 4.16; df = 2, 9; *p* = 0.074), until 35 days post-treatment. At day 35, nymphal mortality for both treatments was significantly (*F* = 7.41; df = 2, 9; *p* = 0.024) higher than the control. When corrected for control using the Sun-Shepard’s formula (39), the nymphal percent mortality (89 ± 3.3) in the fungal treatment was 21% higher than the chemical treatment (68 ± 3.0) 7 days post-treatment. After that time, the percent mortality for both pesticide treatments was similar for the duration of the observation period.

### 3.5. Effects of Treatment on the Occurrence of Enzootic Fungal Species

The mean percent of enzootic entomopathogenic fungi isolated from nymphs per leaf disk varied over time (Table 2). 

When dead nymphs were randomly removed from leaf disks collected pre-treatment (day 0) and then incubated under high humidity, *Aspergillus* sp. occurred 55%, 50% and 40% and *Fusarium* sp. 45%, 45% and 60% of the time from PFR-97, Admire Pro, and control treatments, respectively. In addition, in the Admire Pro treatment plots, 5% of nymphs were infected with *Lecanicillium* sp. On day 14, *Aspergillus* sp., *I. fumosorosea* and *Fusarium* sp. were isolated from 35%, 5% and 60% of the nymphs from PFR-97 treatments prior to the second spray application, respectively. In the control and Admire Pro treatments, 85%, 15%, 0% and 39%, 16%, 45% of the nymphs were infected with *Aspergillus* sp., *P. lilacinum and Fusarium* sp., respectively. At the end of the pilot study nymphs in the PFR-97, Admire Pro and control treatments were infected by *Aspergillus* sp. 55%, 70%, and 65% of the time, and *Fusarium* sp. 30%, 30% and 35% of the time, respectively. Nymphs in the PFR-97 treatment were infected 15% of the time with *Lecanicillium* sp.

The mean number of CFUs removed from leaf disk washes varied considerably per fungal species for each treatment over time (Table 3). 

Overall, the highest number of CFUs days post-application (DPA) for *Aspergillus* sp., *Lecanicillium* sp., *I. fumosorosea*, *P. lilacinum*, *Fusarium* sp., *Penicillium* sp., and *Trichoderma* sp. isolated from leaves collected for all treatments was 190 ± 45, 90 ± 66, 1010 ± 1010, 750 ± 279, 320 ± 135, 80 ± 65, and 10 ± 10, respectively. In the PFR-97 treatment, the number of CFUs for *Aspergillus*, *Lecanicillium*, *P. lilacinum* and *Fusarium* species decreased to zero on 15 DPA, except for *I. fumosorosea* which increased by 16.8 times from 1 DPA. The number of CFUs in the Admire Pro treatment for *Aspergillus* and *P. lilacinum* sp. increased 2.7 and 8 times after 15 DPA compared to 1 DPA, respectively, while the *Fusarium* sp. decreased by 1.5 times; all the other fungal species were not isolated from the leaves collected either 1 or 15 DPA. In the untreated control, the number of CFUs isolated for *Aspergillus* and *P. lilacinum* species decreased by 2 and 4.3 times from leaves collected 15 DPA compared to 1 DPA, respectively, while *P. lilacinum* sp. decreased to zero. No CFUs of *I. fumosorosea*, *Lecanicillium* sp., *Penicillium* sp. and *Trichoderma* sp. were isolated from leaves collected 15 DPA in the untreated plots. At 28 DPA, isolated from leaves collected in the PFR-97 plots, the number of CFUs of *Aspergillus* sp., *P. lilacinum*, *Fusarium* sp. and *Penicillium* sp. increased from 0 at 15 DPA to 20 ± 11, 20 ± 20, 30 ± 20 and 80 ± 65 at 28 DPA, respectively; however, *I. fumosorosea* decreased by 50.5 times to 20 ± 11. From leaves collected in the Admire Pro plots 28 DPA compared to those at 15 DPA, the number of CFUs of *Aspergillus* sp., *P. lilacinum* and *Fusarium* sp. decreased by 6.3, 2.7, and 3.1 times. Also, CFUs of *Lecanicillium* sp., *I. fumosorosea* and *Penicillium* sp. were isolated from leaves collected at 28 DPA. In the untreated control plot, CFUs of all fungal species except *Penicillium* sp. were isolated from leaves collected at 28 DPA. *Trichoderma* sp. CFUs were observed only in the untreated control plots at 28 DPA. 

### 3.6. Isaria Fumosorosea: Ecological Assessment

**Figure 4:**
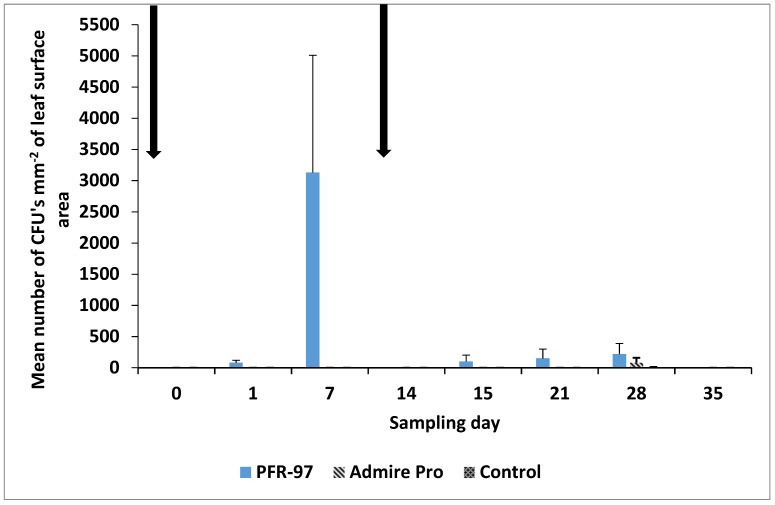
Mean number of colony forming units (CFUs) per mm^2^ of *Isaria fumosorosea* isolated from leaves infested with ficus whitefly on *Ficus benjamina*/treatment. CFUs are based on leaf washes of 10 leaf disks per plot (a total of 40 leaves per treatment); 100 µL of the suspension spread on 5 PDA-dodine selective medium agar plates and then incubated at 25 °C, 100% RH under a 16 h photophase. Error bars represent the mean number of *I. fumosorosea* CFUs ± SEM isolated and arrows indicate when spray application of *I. fumosorosea* was conducted.

The mean number of CFUs mm^−2^ of the leaf surface area for a naturally occurring enzootic population of *I. fumosorosea* was not observed in any of the plots prior to spray (Figure 4).

In the PFR-97 plots, the number of CFUs pre-treatment was 0, and then increased 1-day post-treatment to 81 ± 39 CFUs mm^−2^. After 7 days the number of CFUs mm^−2^ increased >5.5 times to 3130 ± 1880 and on day 14, no CFUs mm^−2^ were isolated from the leaves collected in the PFR-97 plots. The mean number of CFUs mm^−2^ for *I. fumosorosea* was 101 ± 101 the day after the second fungal spray application (day 15), and increased to 150 ± 150 one week after application (day 21). On day 28, the mean number of CFUs isolated from leaf washes in the PFR-97, Admire Pro, and control plots was 220 ± 170, 80 ± 80 and 7 ± 7, respectively. No CFUs mm^−2^ of *I. fumosorosea* were isolated from leaves collected in any of the plots after that time. 

### 3.7. Effect of Treatments on Parasitism Rate of Parasitoids

There was no significant effect of treatment (*F* = 2.69; df = 2, 6; *p* = 0.1466) on parasitism rate between the treatments and control observed on leaves sampled pre-treatment (day 0) (Table 4).

Total mean percent of whitefly nymphs parasitized per sampling day was not significantly different on day 14 (*F* = 3.97; df = 2, 6; *p* = 0.0798) or 35 (*F* = 1.00; df = 2, 6; *p* = 0.4219) amongst treatments for the duration of the study. 

**Figure 5:**
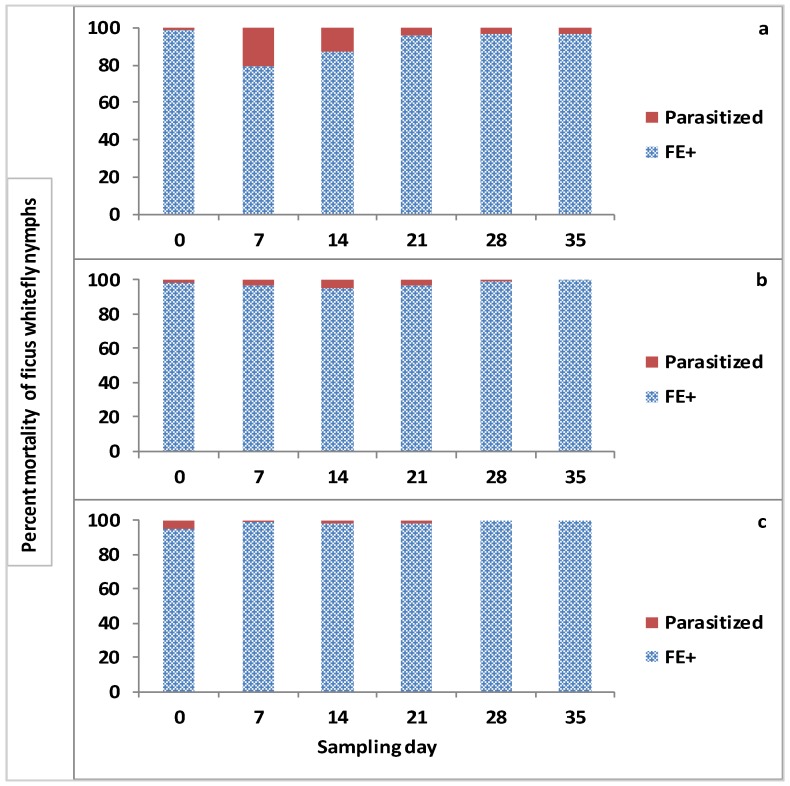
Total percent mortality of the ficus whitefly nymphs caused by fungal entomopathogens plus other biotic and abiotic factors (FE+) and parasitization in the (**a**) control (**b**) PFR-97 and (**c**) Admire Pro treatment plots over time.

The total percentage of mortality due to either infection by EPF plus other factors (biotic: i.e., septicemia, predation and abiotic: i.e., desiccation) and also the nymphs parasitized per sampling day varied over the 35-day observation period for this study (Figure 5).

The total mean percent mortality of the whitefly nymphs on day 7 due to FE+ and parasitization was 79.4 and 20.6, 97.1 and 2.9, and 98.5 and 1.5 for control, PFR-97 and Admire Pro treatment plots, respectively. On day 14, the percent mortality of the nymphs parasitized was higher (12.7%) in the control treatments compared to the fungal (5.2%) and neonicotinoid (1.8%) treatments. On day 21, the total percentage mortality due to parasitization for the control and fungal treatments was similar being 3.8% and 3.1%, which was at least 2× higher than that observed parasitized in the Admire Pro treatment plots. The percent mortality due to parasitization in the control plots remained the same for the rest of the observation period of the pilot study. There was no parasitization of nymphs observed on the leaves collected in the PFR-97 and Admire Pro treatment plots on day 35; therefore, 100% mortality of whitefly nymphs assessed in the treatment plots was due to infection by the fungal entomopathogens present, which included the addition of *I. fumosorosea* propagules on the leaf surfaces plus other factors. Of the fungal entomopathogens, mortality of the whitefly nymphs in the fungal treatment was primarily due to the dual application of PFR containing *I. fumosorosea*. In the neonicotinoid treatment, whitefly nymphal mortality was also due to the toxic lethal effect of the systemic insecticide present inside the leaves.

## 4. Discussion

The fungal spray application with *I. fumosorosea* blastospores had a higher efficacy for managing the whitefly population for the first 7 days, in comparison to the untreated control and the neonicotinoid treatment plots; however, after this time the percent mortality of the ficus whitefly population did not differ significantly between either treatment compared to the control until day 35. Mortality of nymphs after the second application (day 35) in the fungal treatment plots was significantly higher compared to the untreated control, suggesting that two applications of the fungi were compatible with the natural enemies present and helped suppress the whitefly population for 14 days. In the fungal treatment, the blastospore deposition and spray coverage for the initial application was not as uneven on both sides of the leaves as compared to the second spray. The concentration (10^7^ spores mL^−1^) or deposition (~100–400 spores mm^−2^) used in this study was comparable to that used by other researchers for controlling aleyrodid insect pests [22,33,40,41]. In addition, the PFR-97 treatment applied against the ficus whitefly was found to be compatible with its natural biocontrol agents, including the parasitoids *E. protransvena* and *A. bennetti*. This compatibility finding is consistent with other researchers that have studied similar plant-pest/parasitoid-pathogen-predator or multi-trophic interactions with fungal entomopathogens, including *I. fumosorosea* and aleyrodid pests [22,33]. Parasitism of the ficus whitefly by *E. protransvena* and *A. bennetti* did occur in all the treated plots over time. This result suggests that the augmentation of *I. fumosorosea* or a neonicitinoid can be used compatibly with the parasitoids observed for management of the ficus whitefly under field conditions. 

Throughout the duration of the study, a common trend became apparent where the number of parasitized nymphs decreased over time in all the plots. This scenario could be accounted for by having a lower number of nymphs available for *E. protransvena* or *A. bennetti*, to parasitize, due to leaf drop. Gerling et al. [42] indicated that all *Encarsia* sp. parasitize and emerge from the dead 4th instar whitefly hosts but attack mainly the 2nd–4th host instars. In contrast, *A. bennetti* prefers to oviposit in the 1st and 2nd nymphal instars of another whitefly *Bemisia* sp., but can also attack the 3rd and 4th instars [43,44,45]. However, in this study, the different rates of parasitization per nymphal instars of the ficus whitefly that was preferred by each parasitoid was not determined, but only the effect of the enzootic fungal entomopathogens, including the application of *I. fumosorosea* and a neonicotinoid on the overall parasitization by both parasitoids combined. The ratio of parasitization and the preference of nymphal hosts of the ficus whitefly by each parasitoid is unknown and warrants further research. Therefore, due to the lack of older or younger nymphal instars available because leaf drop occurred >14 days after spraying, the overall parasitization rate per treatment plots for the combined parasitoids would naturally decrease over time. In addition, the fungal entomopathogens in both treatment plots compared to the control which increased in total percent mortality of the whitefly nymphs over time would subsequently decrease the amount of susceptible nymphal hosts that were not infected by the fungal entomopathogens. Fransen et al. [26] reported that *E. formosa* could differentiate between greenhouse whitefly nymphs infected with the fungal entomopathogen, *A. aleyrodis* and preferred to oviposit in only healthy uninfected insects when given a choice. All these factors could have contributed to the rapid decrease in the number of nymphs being parasitized in both treatment plots over time.

It is apparent from the current study that both treatments did not have a negative impact on the enzootic entomopathogenic fungal growth and subsequent infection of the ficus whitefly nymphs over time. In fact, throughout this field pilot study, ~95%–100% mortality of the whitefly nymphs assessed in the treatment plots was due to natural causes such as fungal entomopathogens and predators and other factors, with ~5% or less being due to parasitization. The fungal species isolated from the mycosed nymphs were assumed to have caused the mortality of the insect; however, this hypothesis was not confirmed, but warrants further elucidation. From the samples collected in this study, Avery et al. [2] isolated and recorded the following hypocrealean fungi, *I. fumosorosea*, *P. lilacinum*, and *Aspergillus*, *Lecanicillium*, and *Fusarium* species from ficus whitefly, *S. simplex* nymphs. In addition, *Penicillium* and *Trichoderma* fungal species were also identified and recorded from the leaf wash samples from this study. Torres-Barragán et al. [15] isolated *Aspergillus*, *Penicillium*, *Paecilomyces*, *Lecanicillium*, *Aschersonia*, and *Fusarium* species from insects collected, including whiteflies from the agricultural area in Mexico. In another study, Scorsetti et al. [46] isolated *I. fumosorosea*, *I. javanica* and *Lecanicillium* species from whiteflies collected on organic and conventional horticultural crops in Argentina. 

In this pilot study, two fungal species that had the highest percent occurrence infecting the ficus whitefly nymphs over time was *Aspergillus* and *Fusarium*. These fungal species are most likely saprophytic; however, a few authors have evaluated them as potential biocontrol agents for controlling various whitefly insect pest species [47,48,49,50]. Whether these fungal species were entomopathogenic to the whitefly is unknown and requires further testing. The other fungi, *I. fumosorosea*, and *Lecanicillium* species, not isolated as often from the whitefly nymphs, are common entomopathogenic fungi used as fungal biopesticides [51] for controlling whitefly in many crop systems [40,41,46,52]. In addition, *I. fumosorosea* and *Lecanicillium* sp. are also compatible with predators and parasitoids used in IPM programs for control of whitefly insect pests [22,24,31,32,33]. Another fungal species, *P. lilacinum*, which is more commonly found in the soil and used for the biological control of nematodes, was recorded from the leaf wash samples. Although this is a rare occurrence for this fungus to infect the ficus whitefly, recently *P. lilacinum* has demonstrated much potential as a biocontrol agent of the greenhouse whitefly [41,53]. 

Although *Aspergillus*, and *Fusarium* species were isolated from the majority of dead insects removed from the collected leaves, *Penicillium* and *Trichoderma* species were only isolated and identified from leaf wash plated samples. In addition, *I. fumosorosea* CFUs were counted on leaf wash sample plates after spraying, but the increase after the second application was much less compared to the first. In a concurrent laboratory study, Avery et al. (unpublished data) found in vitro that *Aspergillus*, *Fusarium*, *Penicillium* and *Trichoderma* species were antagonistic and/or pathogenic to *I. fumosorosea*. Therefore, the germination and fungal growth of *I. fumosorosea* could have been inhibited by the presence of secondary plant compounds produced by *F. benjamina* plants which have antimicrobial properties [54,55,56]. Based on the in vitro bioassay results and possible antimicrobial properties, we speculate that due to the interspecific and intraspecific competition with the antagonistic fungal pathogens present on the leaf phylloplane, that the fungal spores or propagules of *I. fumosorosea* may have been inhibited from germination and growth, which subsequently would affect the efficacy of this entomopathogenic fungus contained in PFR-97 after application. However, these hypotheses need further evaluation to confirm the actual event. Competition between entomopathogenic fungal species is now considered an important biotic aspect to understand in order to increase the efficacy of biopesticides for managing arthropod pests [19,20,57]. 

Based on the CFUs isolated, it was evident that after the *I. fumosorosea* blastospores were applied, their numbers increased significantly from 0 to 81 CFUs mm^−2^ on day 1 to >5.5 times to 3130 on day-7 post-treatment. It has been observed that *I. fumosorosea* blastospores after application and contact to a leaf surface, will germinate and produce conidia in ~7 days under high humidity (>70% RH) and temperatures (~25 °C) [58]. In addition, the environmental conditions i.e., mean temperatures and RH which remained at 24.4 °C and 75.6% RH, respectively throughout this 35-day study, were conducive for the growth of *I. fumosorosea* [27]. In the second application, the CFUs mm^−2^ were slightly higher (101) as the initial application, but increased only ~1.5 times to 150 on day-7 post-treatment. However, the mean number of *I. fumosorosea* CFUs mm^−2^ isolated from the leaf washes diminished to zero 14 days and 21 days post-treatment for 1st and 2nd applications, respectively. The lack of CFUs mm^−2^ being observed may be accounted for by any of the following or a combination: (1) increased rainfall during that time, (2) biodegradation of conidia due to exposure to ultra violet rays or high diurnal temperatures [59,60], (3) intra- and interspecific competition with other fungal pathogens [57], (4) potential inhibition of fungal germination and hyphal growth due the presence of secondary compounds on the leaf surface [54,55,56], (5) lack of nymphal density to trap the spores from being washed off the waxy leaf surface, and (6) removal of inoculum due to extreme leaf drop. 

It is interesting that 14 days after the second application, *I. fumosorosea* CFUs mm^−2^ were present in all the treatments. This suggests that the conidia were being spread around to all the treatments by wind, rain or other organisms present in the ecosystem e.g. pest insects, predators and parasitoids. Avery et al. [61] found that the fungal blastospores and conidia of *I. fumosorosea* could be spread from one leaf to another by a single adult Asian citrus psyllid, *Diaphorina citri*. Therefore, it is possible that if whitefly adults emerged and became contaminated with conidia on the leaf surface, they could potentially disperse and deposit *I. fumosorosea* spores and/or propagules on to other plants. The ladybird beetles, *H. axyridis* and *C. coeruleus* observed on the PFR-97 plots could have dispersed the fungal spores as they searched for whiteflies in the other treatment plots. For instance, Sànchez Barahona et al. [35] observed that the ladybird beetle, *Thalassa montezumae* Mulsant was able to disperse fungal spores or propagules of *I. fumosorosea* as it roamed and fed on the green croton scale insects infesting croton plants previously sprayed with PFR-97. Both adult whiteflies and their specific parasitoids may also have been involved in horizontal transmission of the spores or propagules. Fransen and van Lenteren [26] found that the parasitoid, *E. formosa* was responsible for transmitting the fungal spores of *Aschersonia aleyrodis* after probing the greenhouse whitefly nymphs to a limited extent. This hypothesis is interesting and warrants further research. The use and role of non-target organisms in dispersing entomopathogenic fungi in integrated pest management systems is reviewed in Skinner et al. [62].

## 5. Conclusions

Based on the current study, it can be concluded that the endemic population of predators, parasitoids and enzootic population of fungal entomopathogens must be considered as part of a multi-trophic ecosystem, and that there may be an interaction after the application of any pesticide. The fungal biopesticide, PFR-97 and neonicotinoid, Admire Pro were compatible overall on the natural enemies and more effective in managing the invasion of the ficus whitefly, *S. simplex* compared to the untreated control. Therefore, it is important to assess the long-term impact that the application of any pesticide will have on the ecosystem when managing this pest; especially, the ecological impact on all the natural enemies, which includes the enzootic fungal entomopathogens. Although the *Ficus* hedge is a man-made ecosystem, it is still very important that this ecological concept be considered when determining the best long-term, sustainable strategy employed by the homeowner for managing the ficus whitefly. Therefore, in the landscape it is extremely important to use the appropriate insecticides, methods, and timing in order to get the best control with the least amount of detriment to the natural enemies or the environment.

## Figures and Tables

**Table 1:** Insect pests and natural enemies observed on a residential *Ficus benjamina* hedge *.

Category	Order	Family	Scientific Name	Common Name	Observation
Insect pests	Hemiptera	Aleyrodidae	*Singhiella simplex*	ficus whitefly	feeding on leaves
	Hemiptera	Aleyrodidae	*Tetraleurodes fici*	whitefly	feeding on leaves
	Thysanoptera	Phlaeothripidae	*Gynaikothrips uzeli*	weeping ficus thrips	in leaf galls
Natural Enemies	Hymenoptera	Aphelinidae	*Encarsia protransvena*	parasitoid	parasitized nymphs
	Hymenoptera	Platygasteridae	*Amitus bennetti*	parasitoid	parasitized nymphs
	Coleoptera	Coccinellidae	*Harmonia axyridis*	Asian lady beetle	roaming on leaves
	Coleoptera	Coccinellidae	*Curinus coeruleus*	metallic blue lady beetle	roaming on leaves
	Neuroptera	Chrysopidae	*Chrysopid* sp.	green lacewing	eggs, larvae on leaves
	Hypocreales	Clavicipitaceae	*Isaria fumosorosea*	fungal species	leaf surface, nymphs
	Hypocreales	Ophiocordycipitceae	*Purpureocillium lilacinum*	fungal species	leaf surface, nymphs
	Hypocreales	Plectosphaerellaceae	*Lecanicillium* sp.	fungal species	leaf surface, nymphs

* Adapted and revised from Avery et al. [2].

**Table 2:** Mean percent occurrence of fungal species infecting ficus whitefly nymphs infesting *Ficus benjamina* leaves on various sampling days per treatment.

	Mean % Occurrence of Each Fungal Species on Ficus Whitefly Nymphs/Treatment/Sampling Day ^a^								
		0			14			35	
Fungal Species	PFR-97	Admire Pro	Control	PFR-97	Admire Pro	Control	PFR-97	Admire Pro	Control
*Aspergillus* sp.	55	50	40	35	39	85	55	70	65
*Lecanicillium* sp.	0	5	0	0	0	0	15	0	0
*Isaria fumosorosea ^b^*	0	0	0	5	0	0	0	0	0
*Purpureocillium lilacinum*	0	0	0	0	16	15	0	0	0
*Fusarium* sp.	45	45	60	60	45	0	30	30	35

^a^ Numbers based on 20 nymphs randomly chosen and removed from 40 semi-desiccated leaves. Fungal isolates identified after incubating nymphs in water agar plates at 25°C at 100% RH under a 16 h photophase; ^b^ *I. fumosorosea* at day 14 was not determined if enzootic or resulting from the 1st PFR-97 spray application.

**Table 3:** Fungal species isolated from ficus whitefly nymphs and leaves of *Ficus benjamina* various days post-application.

	Mean Number of Colony Forming Units (CFUs) ± SEM (×10^1^)/Days Post-Application/Treatment ^a^								
	1			15			28		
	PFR-97	Admire Pro	Control	PFR-97	Admire Pro	Control	PFR-97	Admire Pro	Control
Fungal species ^b^									
*AS*	13 ± 0.4	7 ± 2.5	2 ± 1.2	0	19 ± 4.5	1 ± 0.1	2 ± 1.1	3 ± 1.3	9 ± 2.4
*LE*	9 ± 6.6	0	0	0	0	0	0	1 ± 0.5	9 ± 6.1
*IF* ^c^	6 ± 2.6	0	0	101 ± 101.0	0	0	2 ± 1.1	1 ± 0.5	5 ± 5.0
*PL*	75 ± 27.9	1 ± 0.5	34 ± 13.0	0	8 ± 6.6	8 ± 3.5	2 ± 2.0	3 ± 2.2	31 ± 1.1
*FU*	32 ± 13.5	51 ± 15.2	3 ± 1.3	0	22 ± 10.0	0	3 ± 2.0	7 ± 1.1	4 ± 1.7
*PN*	0	0	0	0	0	0	8 ± 6.5	1 ± 1.0	0
*TR*	0	0	0	0	0	0	0	0	1 ± 1.0

^a^ Mean number of CFUs is based on 40 leaf disks per treatment, 10 per plot. Fungal isolates were identified after spreading 100 µL on 5 PDA-dodine selective medium agar plates sealed and incubated at 25 °C, under a 16 h photophase; ^b^ *AS* = *Aspergillus* sp.; *LE* = *Lecanicillium* sp.; *IF* = *Isaria fumosorosea; PL* = *Purpureocillium lilacinum*; *FU* = *Fusarium* sp.; *PN* = *Penicillium* sp.; *TR* = *Trichoderma* sp; ^c^ PFR-97 20% WDG formulated product containing blastospores of *I. fumosorosea* was sprayed on day 0 and 14.

**Table 4:** Effect of treatments on the total mean percent of ficus whitefly nymphs per plot parasitized observed on *Ficus benjamina* leaves.

	Total Mean % of Ficus Whitefly Nymphs Parasitized/Sampling Day ^a^		
Treatment	0	14	35
PFR-97	40 ± 21.3 ^a^	15 ± 11.1 ^a^	0 ± 0.0 ^a^
Admire Pro	36 ± 21.8 ^a^	4 ± 3.8 ^a^	0 ± 0.0 ^a^
Control	14 ± 12.2 ^a^	39 ± 20.5 ^a^	13 ± 12.5 ^a^

^a^ Percent mean are based on observing 40 leaves per treatment, 10 leaves per plot. Data was square root (*n* + 0.01) arcsine transformed to remove zeros prior to analysis; untransformed data are presented in table. The total mean percentage of ficus whitefly nymphs parasitized in either treatment was not significantly different per sampling day (Tukey’s HSD test, *p* > 0.05).
